# IL-18 Mediates Vascular Calcification Induced by High-Fat Diet in Rats With Chronic Renal Failure

**DOI:** 10.3389/fcvm.2021.724233

**Published:** 2021-11-25

**Authors:** Yinyin Zhang, Kun Zhang, Yuling Zhang, Lingqu Zhou, Hui Huang, Jingfeng Wang

**Affiliations:** ^1^Cardiology, Sun Yat-sen Memorial Hospital, Guangzhou, China; ^2^Cardiology, The Eighth Affilliated Hospital, Sun Yat-sen University, Shenzhen, China

**Keywords:** IL-18, vascular calcification, chronic renal failure, high-fat diet, MAPK

## Abstract

**Objective:** Vascular calcification (VC) is an important predictor of cardiovascular morbidity and mortality in patients with chronic renal failure (CRF). It is well-known that obesity and metabolic syndrome (OB/MS) predicts poor prognosis of CRF patients. However, the influence of OB/MS on VC in CRF patients isn't clear. IL-18 mediates OB/MS-related inflammation, but whether IL-18 is involved in OB/MS -mediated VC in CRF patients hasn't been studied. In this study, it was explored that whether OB/MS caused by high-fat diet (HFD) can affect the level of serum IL-18 and aggravate the degree of VC in CRF rats. Furthermore, it was studied that whether IL-18 induces rat vascular smooth muscle cells (VSMCs) calcification by activating the MAPK pathways.

**Approach:** The rats were randomly assigned to the sham-operated, CRF and CRF + HFD groups. CRF was induced by 5/6 nephrectomy. Serum IL-18 levels and aortic calcification indicators were compared in each group. Primary rat VSMCs calcification were induced by β-glycerophosphate and exposed to IL-18. VSMCs were also treated with MAPK inhibitors.

**Results:** The weight, serum levels of hsCRP, TG and LDL-C in CRF + HFD group were significantly higher than those in sham-operated and CRF groups (*p* < 0.05). Compared with the sham-operated group, the calcium content and the expression of BMP-2 of aorta in CRF and CRF + HFD groups were significantly increased (*p* < 0.05). Moreover, the calcium content and the expression of BMP-2 of aorta in CRF + HFD group was significantly higher than those in CRF group (*p* < 0.05). And the serum IL-18 level was positively correlated with aortic calcium content. It was also found that p38 inhibitor SB203580 can suppress the VSMCs calcification and osteoblast phenotype differentiation induced by IL-18. But the JNK inhibitor SP600125 can't suppress the VSMCs calcification and osteoblast phenotype differentiation induced by IL-18.

**Conclusions:** These findings suggest that obesity-related inflammation induced by high-fat diet could exacerbate VC in CRF rats. Furthermore, serum IL-18 level had a positive correlation with the degree of VC. It is also found that IL-18 promoted osteogenic differentiation and calcification of rat VSMCs via p38 pathway activation.

Vascular calcification (VC) is a pathological process which is increased by age and aggravated in chronic diseases including chronic kidney disease (CKD), hypertension, diabetes mellitus and bone-mineral disorders (1, 2). VC is a common vascular complication in patients with chronic renal failure (CRF), which induce a series of adverse effects on hemodynamics, and is an important factor responsible for the increase in the morbidity and mortality of cardiovascular diseases in patients with CRF (3, 4). VC is believed to be an actively regulated process which is similar with bone formation and is initiated by phenotype change of vascular smooth muscle cells (VSMCs) to osteoblast-like cells (5).

Inflammation is known to be a key factor in promoting the formation of VC, but the exact pathological mechanism of inflammation-mediated VC is not yet fully understood (6, 7). IL-18 (interleukin-18) is a proinflammatory cytokine that belongs to the IL-1 superfamily and is produced by macrophages and other cells, including VSMCs (8). Our previous study had found that IL-18 is an important inflammatory factor that induces VSMCs calcification (7). Many studies have found that serum IL-18 levels in patients with CRF are significantly higher than those with normal renal function (9, 10). It is well-known that VC is the most common vascular complication in patients with CRF, but whether IL-18 is involved in the development of VC in patients with CRF is not clear.

Energy-dense food with a high fat content is identified to implicated in the pathogenesis of obesity and metabolic syndrome (OB/MS) (11). It is well-known that obesity can cause kidney damage, and its mechanism is more complicated. Current research believes that obesity damages the kidneys through four ways: hypertension, hyperglycemia, hyperlipidemia, and hyperuricemia. And at the same time, the four factors affect each other, forming a vicious circle (12, 13). Adipose tissue is also an important endocrine system of the body. It can produce a variety of adipokines, including leptin, adipocytokines, adiponectin, etc., as well as tumor necrosis factor-α, monocyte chemotactic factor and angiotensin II. These substances may have a direct effect on the occurrence and development of CRF (14). VC is an important vascular complication in patients with CRF, which indicates increased cardiovascular and death risk. Our previous research suggested that in subjects without CRF, those with OB/MS had higher incidence VC and the OB/MS-related inflammation might be involved in regulating the formation of VC (15, 16). However, the influence of OB/MS on VC in patients with CRF has not been studied in detail. A large amount of research evidence confirms that IL-18 mediates OB/MS-related inflammation (17). However, whether IL-18 is involved in the pathogenic mechanism of OB/MS -mediated VC in patients with CRF has not yet been studied.

The mitogen-activated protein kinase (MAPK) pathway is responsible for conveying information about the extracellular environment to the cell nucleus and is known to play a critical role in osteoblast differentiation and mineralization (18). JNK and p38 are the main MAPK signaling pathways. At present, studies suggest that the MAPK pathway is involved in the development of VC (19, 20). Studies have also proved that IL-18 can cause biological effects by activating MAPK pathway (21, 22). Therefore, we speculate that IL-18 may promote VSMCs calcification and osteoblast phenotype differentiation by activating the MAPK signaling pathway.

This study was based on a rat model of CRF to observe whether OB/MS caused by high-fat dietary (HFD) can affect the level of serum IL-18 in rats, aggravate the degree of VC, and initially reveal the relationship between IL-18 and OB/MS-related VC in CRF rats. Furthermore, we explore whether IL-18 induces rat VSMCs calcification and osteoblast phenotype differentiation by activating the MAPK pathways, so as to further clarify the mechanism of IL-18 inducing VC.

## Materials and Methods

### Animal Model and Grouping

The animal experiments were approved by the Committee on Ethics of Animal Experiments and conducted in accordance with the Guidelines for Animal Experiments, Sun Yat-sen University and the Guide for the Care and Use of Laboratory Animals published by the US National Institutes of Health (NIH Publication No. 85-23, revised 1996).

Male Sprague-Dawley rats with an average body weight of 200–250 g were used in this study. All the animals were housed in an environmentally controlled room at 24 ± 1°C with a 12 h light/dark cycle and fed with tap water. The rats were randomly assigned to the CRF and sham-operated groups. CRF was induced by 5/6 nephrectomy (5/6 Nx) with surgical excision of two-thirds of the left kidney, followed by the complete right nephrectomy 1 week later. Sham-operated group underwent similar surgical procedures but with only removal of the renal envelope. The operations were carried out under general anesthesia (pentobarbital sodium, 50 mg/kg ip) using strict hemostasis and aseptic techniques. The 5/6 Nx rats were randomly divided into CRF and CRF + high fat diet (HFD) groups. The sham-operated and CRF groups were fed a standard laboratory diet with a total fat content of 4.3%. The CRF + HFD group were fed a HFD with a total fat content of 34.9%. Six animals were included in each group. Six months later, rats were sacrificed and serum levels of IL-18 were measured with commercially available kits (Ab Frontier). The aortas were dissected for calcium deposition assay, von Kossa and Alizarin red S staining, RNA and protein extraction.

### Cell Culture

Primary aortic VSMCs of 2-month-old male Sprague–Dawley rats were obtained as described previously (7) and maintained in the high glucose (4.5 g/L) Dulbecco's modified Eagle's medium (DMEM, Gibco) containing 10% fetal bovine serum (FBS), 100 U/ml penicillin and 100 mg/ml streptomycin at 37°C in a humidified atmosphere containing 5% CO_2_. The cells at passages 4–8 were used for the experiments. Each experiment was repeated for at least three times. VSMCs calcification was induced by calcifying medium, DMEM containing 10% FBS, 10 mM sodium pyruvate, 100 U/ml penicillin, 100 mg/ml streptomycin and 10 mM β-glycerophosphate (β-GP, Sigma) for 14 days with medium changes every 2 days 4. After using IL-18 (R&D) to interfere with rat VSMCs for 2, 5, 10, 15, and 30 min, the phosphorylation and non-phosphorylation protein expression levels of two MAPK pathways including p38 and JNK were detected by Western blot. Rat VSMCs were pre-incubated with p38 inhibitor SB203580 (Sigma) at a final concentration of 10 μmol/L for 2 h, and then treated with β-GP (10 mmol/L) or IL-18 (100 ng/ml) + β-GP (10 mmol/L) for 14 days, respectively. Rat VSMCs were also pre-incubated with JNK inhibitor SP600125 (Sigma) at a final concentration of 10 μmol/L for 30 min, and then treated with β-GP (10 mmol/L) or IL-18 (100 ng/ml) + β-GP (10 mmol/L) for 14 days, respectively.

### Serum Analyses

Blood of mice was collected from the caudal vein and serum concentrations of creatinine (Cr), calcium (Ca), phosphate (P), total cholesterol (TC), triglycerides (TG), high-density lipoprotein cholesterol (HDL-C), low-density lipoprotein cholesterol (LDL-C) and high-sensitivity C-reactive protein (hsCRP) were measured by a standardized and certified program with an automatic biochemical analyzer (7170A, HITACHI, Japan).

### Measurement of Systolic and Mean Blood Pressures

Systolic blood pressure (SBP) and mean blood pressure (MBP) were obtained by a tail-cuff measurement (BP-98A, Softron, Tokyo, Japan). Conscious rats were placed in a restrainer with an electrical warming pad for 20-min and trained for 1 week before testing. In order to avoid variations of the SBP and MBP, all measurements were carried out between 8 and 11 am. At least three measurements of each rat were taken at 2 min intervals and the mean values of MBP and SBP were calculated.

### Alizarin Red S Staining

Alizarin red S staining method was used to determine the calcification of rat aortas and VSMCs (7). The paraffin sections of the rat aortas were deparaffinized twice with xylene. And then the aortic slices were subjected to gradient concentration of ethanol and pure water each time for 5 min. After that, the sections were washed with phosphate buffered saline (PBS, Gibco) and stained with 2% Alizarin (sigma) for 30 min. Finally, the sections were washed with PBS before air-dry, and then sealed with neutral gum. VSMCs were with PBS and then fixed with 4% paraformaldehyde. After that, the cells were washed with PBS and exposed with 2% Alizarin Red S for 10 min, and then observed under the microscope. Positively stained cells displayed a red color.

### Von Kossa Staining

The rat aorta and VSMCs slides were fixed with ice acetone at −20°C and washed with PBS. Von Kossa staining was performed as described previously (23). Five percentage silver nitrate solution was added to the slides. Discard the silver nitrate solution and add 1 ml of 5% sodium thiosulfate solution. Back staining with 1% basic fuchsin for 10 s. The slides are dehydrated with anhydrous. Finally, Seal the film with neutral gum and observe the calcium nodules under a light microscope.

### Hematoxylin-Eosin Staining of Rat Aorta

Paraffin sections are deparaffinized with xylene, washed with water after gradient ethanol. After hematoxylin staining for 5 min, the sections were washed with water for 1 min. Then, the sections were differentiated with l% hydrochloric acid alcohol and stained with saturated lithium carbonate. Finally, the sections were stained with eosin, then dehydrated and sealed.

### Quantification of Calcium Deposition

Quantification of VSMCs and aortic calcium deposition was performed as described previously (24). VSMCs were collected and dissolved in 2 mol/L HNO_3_ overnight. Thereafter, VSMCs were re-dissolved with a blank solution (27 nmol/L KCl, 27 μmol/L LaCl_3_ in de-ionized water). The calcium deposition of VSMCs was measured by an atomic absorption spectrophotometer at 422.7 nm (Hitachi, Z-5000). Calcium deposition of VSMCs was normalized by protein concentration. Take the rat aorta (10–20 mg) and dry it thoroughly at 80°C, and weigh. Add 2 mol/L concentrated nitric acid to digest for 24 h, put the digestion tube into the automatic control electric heating digester to digest until all the acid is volatilized. After cooling, reconstitute it with deionized water containing 27 nmol/L KCl and 27 μmol/L LaCl_3_ overnight. Then add 1% strontium chloride and measure the optical density value of each tube at 422.7 nm wavelength with an atomic absorption spectrophotometer. Repeat the measurement for each sample three times and take the average value, and then convert it into the calcium content of the tissue (μmol/gdw).

### Alkaline Phosphatase Activity Assay

The cells were washed with PBS and treated with 1% Triton X-100 in 0.9% NaCl. After centrifugation at 12,000 rpm at 4°C for 10 min, the supernatants were harvested to detect for alkaline phosphatase (ALP) activity with the use of ALP assay kit (Jiancheng Bioengineering Co., Nanjing, China). ALP activity was measured colorimetrically as thehydrolysis of p-nitrophenyl phosphate and the results were normalized to the levels of total protein.

### Immunohistochemistry

For the detection of bone morphogenetic protein-2(BMP-2), immunohistochemical staining was performed. Rat aortas were embedded in paraffin blocks for immunohistochemical staining. Endogenous peroxidase activity was blocked by 3% H_2_O_2_. Sections were incubated with anti-BMP-2 antibody (dilution 1:250, Santa Cruz, California, US) overnight at 4°C, then washed with PBS. Thereafter, sections were incubated with secondary antibody of anti-horseradish peroxidase (HRP) (dilution 1:50, Santa Cruz, California, US) at room temperature for 30 min, then washed again three times with PBS prior to staining with 3, 3′-diaminobenzidine (DAB) at room temperature for 4 min. BMP-2 protein expression was visualized as brown deposits using a microscope.

### Quantitative Real-Time Polymerase Chain Reaction

Trizol and liquid nitrogen were added to every 1 cm rat aorta. Then the tissue was grinded into powder and centrifuged at 1,2000 g at 4°C for 5 min. And the supernatant was aspirated and transferred into a new EP tube. Total RNA from VSMCs and rat aortas was isolated by the Trizol method (TaKaRa, Japan) and was reverse-transcribed with the PrimeScript RT reagent kit (TaKaRa, Japan) following the manufacturer's recommended protocol (7). Amplification reactions were set up in 20 μl reaction volumes containing amplification primers and SYBR Premix Ex Taq TM II (Takara, Japan). One μl cDNA was used in each amplification reaction. Preliminary experiments were carried out to optimize primer concentrations. The PCR primers were as follows: BMP-2, forward 5′-ACCG TGCTCAGCTTCCATCAC-3′ and reverse5′-CTATTTCCCAAAGCTTCCTGCAT TT-3′; GAPDH, forward 5′-GGCACAGTCAAGGCTGAGAATG-3′ and reverse5′-ATGGTGGTGAAGACGC CAGTA-3′. Each sample was run in triplicates and each experiment was repeated at least once. Amplification data were analyzed using the LightCycler 480 real-time PCR instrument (Roche, Germany). Quantification was performed using the ΔΔCt method. The results were normalized to GAPDH and expressed as percentage of controls.

### Western Blot Analysis

The rat aortas were treated with RIPA lysis buffer (Beyotime, Haimen, China) and comminuted with homogenizer on ice. VSMCs were washed twice with cold PBS and treated with RIPA lysis buffer on ice for 30 min. After centrifugation at 12,000 rpm at 4°C for 10 min, the supernatants of the aortic homogenate and VSMCs were harvested to determine the protein expression. The protein samples were mixed with the loading buffer and boiled at 95°C for 5 min. The boiled samples were separated on the SDS–polyacrylamide gels, and the proteins were transferred to the polyvinylidene difluoride (PVDF) membranes. The PVDF membranes were incubated in a blocking buffer containing 5% (w/v) bovine serum albumin (BSA). The blots were then incubated with primary antibodies: anti-total p38 antibody, anti-phospho-p38 antibody, anti-total ERK1/2 antibody, anti-phospho-ERK1/2 antibody, anti-total JNK antibody, anti-phospho-JNK antibody, anti-GAPDH antibody (dilution: 1:1000, Cell signaling technology, Danvers, US), anti-BMP-2 antibody (dilution: 1:300, Santa Cruz, California, US) in TBST containing 5% (w/v) BSA (antibody buffer) overnight at 4°C. The members were then washed and incubated with the horseradish peroxidase-linked secondary antibody (dilution: 1:1000, Cell signaling technology, Danvers, US) and then visualized with the enhanced chemiluminescence (Thermo Fisher Scientific, Waltham, US). The bands were analyzed semi-quantitatively.

### Statistical Analysis

Each experiment was repeated three times independently. Data were expressed as means ± SD. The results were compared with one-way ANOVA followed by Student-Newman-Keuls test for *post-hoc* comparison among more than two groups. Pearson's correlation analysis was used to analyze the relationship between serum IL-18 levels and aortic calcium content. All statistical analyses were performed using the software SPSS 17.0. For all statistical tests, two-tailed *P*-value < 0.05 indicated the statistical significance of the results.

## Results

### Comparison of Weight and Serum Biochemical Indexes Among CRF, CRF + HFD and Sham-Operated Rats

The weight and biochemical indexes of rats in three groups are shown in Table 1. Compared with sham-operated rats, CRF and CRF + HFD rats had higher serum levels of Cr, P and hsCRP (*p* < 0.05) and lower level of Ca (*p* < 0.05). And the weight, serum levels of TG and LDL-C in CRF + HFD rats were significantly higher than sham-operated and CRF rats (*p* < 0.05). Furthermore, the serum level of hsCRP in CRF + HFD rats significantly higher than CRF rats (*p* < 0.05).

**Table 1:** Comparison of body weight and serum biochemical indexes of rats in each group.

	**Sham-operated**	**CRF**	**CRF + HF**
Weight (g)	360 ± 10	334 ± 37.2	401 ± 40.8^#^^*^
Ca (mmol/L)	2.52 ± 0.40	1.92 ± 0.14^#^	1.92 ± 0.22^#^
P (mmol/L)	1.07 ± 0.01	1.72 ± 0.21^#^	1.75 ± 0.26^#^
Cr (μmol/L)	56.67 ± 4.73	104.31 ± 14.44^#^	110.47 ± 19.01^#^
HsCRP (mg/L)	4.45 ± 0.62	8.29 ± 1.25^#^	9.91 ± 1.81^#^^*^
CHOL (mmol/L)	2.10 ± 0.34	2.09 ± 0.35	2.38 ± 0.51
TG (mmol/L)	0.64 ± 0.10	0.58 ± 0.13	1.41 ± 0.45^#^^*^
LDL-C (mmol/L)	0.39 ± 0.06	0.34 ± 0.10	0.49 ± 0.17^#^^*^
HDL-C (mmol/L)	1.00 ± 0.20	0.94 ± 0.16	0.91 ± 0.18

* *p < 0.05 vs. CRF group;*

# *p < 0.05 vs. sham-operated group*.

### Comparison of the Blood Pressure of Rats in Each Group

**Figure 1:**
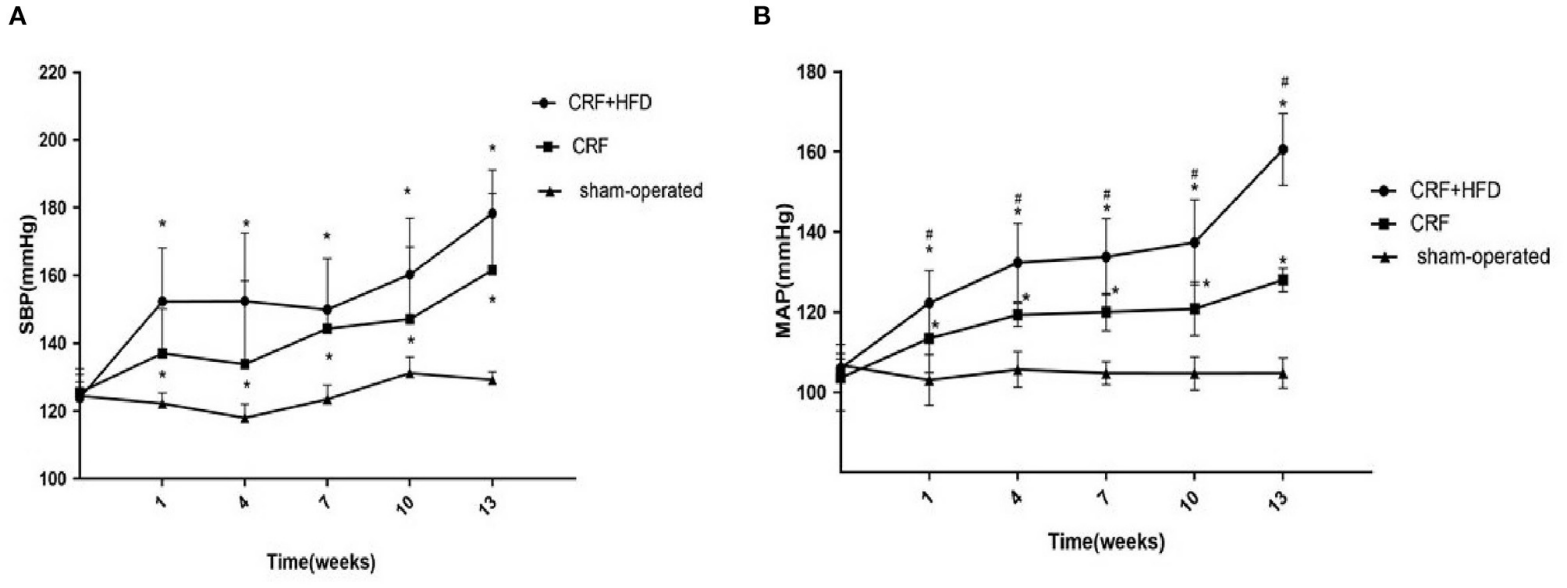
Comparison of systolic blood pressure **(A)** and mean arterial pressure **(B)** of rats in each group. Six rats were included in each group. *P < 0.05 vs. sham-operated group; ^#^*P* < 0.05 vs. CRF group. CRF, chronic renal failure; HFD, high fat diet; MAP, mean arterial pressure; SBP, systolic blood pressure.

The blood pressure of the rat tail was measured before the operation and 1, 4, 7, 10, and 13 weeks after the operation. The results showed (Figures 1A,B) that the systolic blood pressure (SBP) and mean arterial pressure (MAP) in the CRF and CRF + HFD groups showed an upward trend after surgery, but the SBP and MAP in the sham-operated group did not change significantly. From the first week after surgery, the SBP and MAP in CRF and CRF + HFD group were significantly higher than those in the sham-operated group (*p* < 0.05). From the first week after surgery, the MAP in the CRF + HFD group was significantly higher than that of the CRF group (*p* < 0.05), while the SBP in the CRF + HFD group was not significantly different from that of the CRF group (*p* > 0.05).

### Comparison of Calcium Content in the Aorta of Rats in Each Group

**Figure 2:**
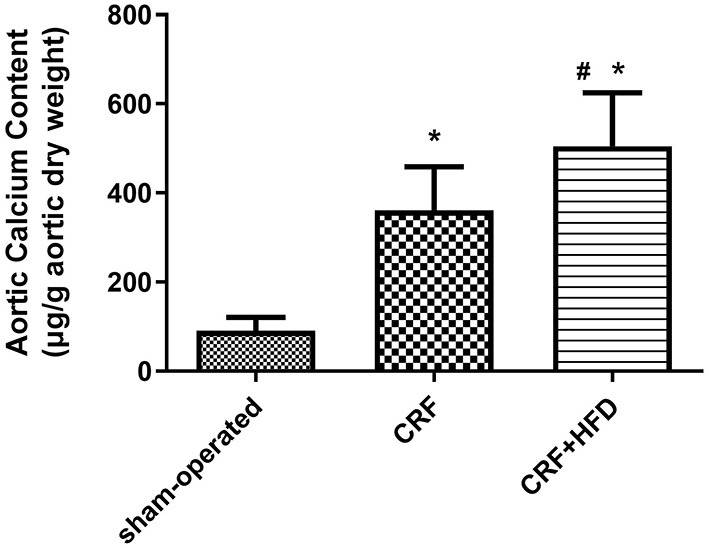
Comparison of calcium content in the aorta of rats in each group. Six rats were included in each group. *P < 0.05 vs. sham-operated group; ^#^*P* < 0.05 vs. CRF group. CRF, chronic renal failure; HFD, high fat diet.

Take the aorta of rats in each group 6 months after operation for the detection of calcium content. The results are shown in Figure 2. Compared with the sham-operated group, the calcium content of the aorta in the CRF and CRF + HFD groups were significantly increased (360.68 ± 98.48, 504.82 ± 120.30 vs. 91.17 ± 29.63, *p* < 0.05). Moreover, the calcium content of aorta in the CRF + HFD group was significantly higher than that in the CRF group (504.82 ± 120.30 vs. 360.68 ± 98.48, *p* < 0.05).

### HE and Calcification Staining of Rat Aortas in Each Group

**Figure 3:**
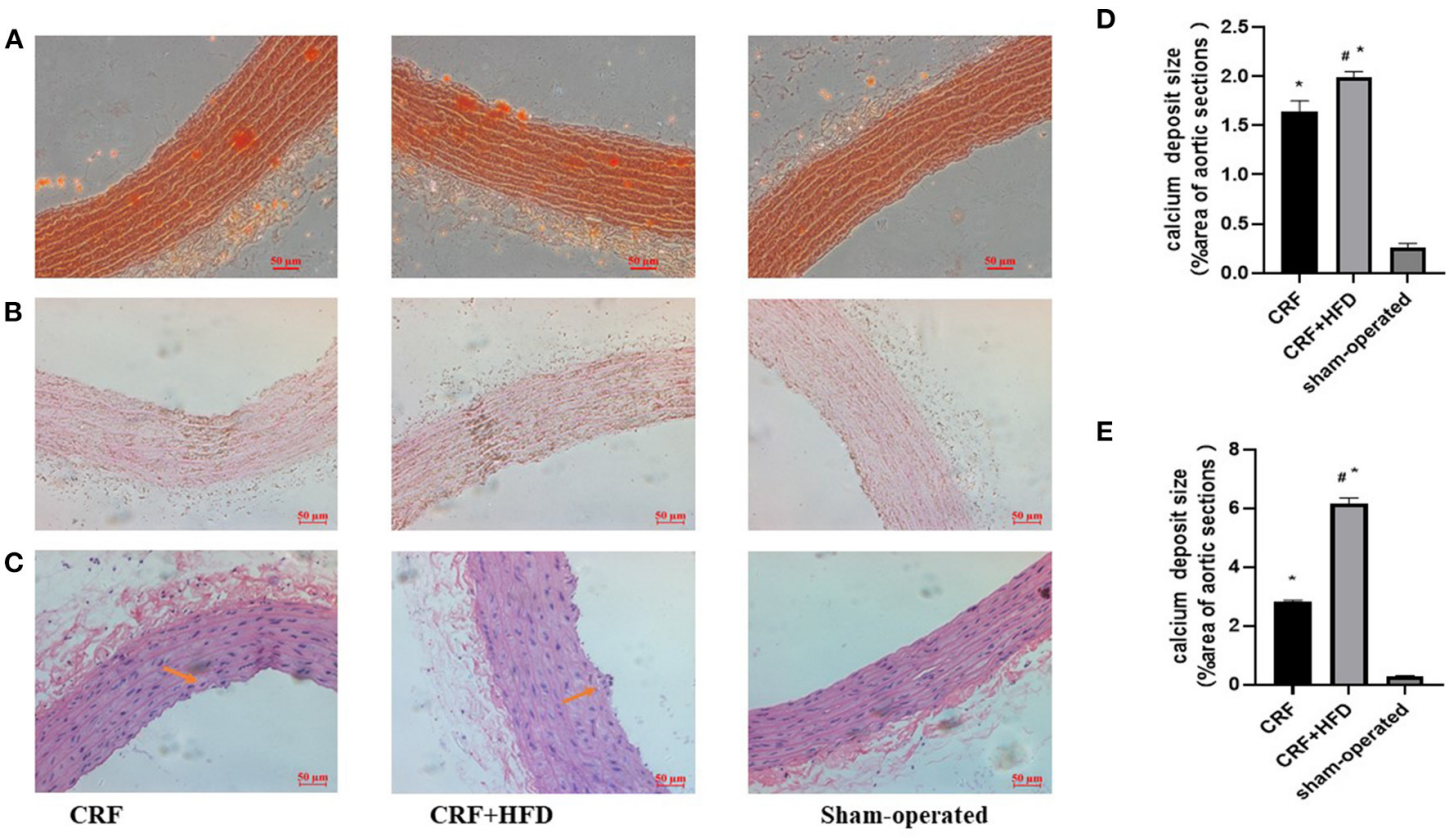
Staining of rat aorta in each group. **(A)** Alizarin red staining of rat aorta in each group. **(B)** Von Kossa staining of rat aorta in each group. **(C)** staining of rat aorta in each group. **(D)** Quantification of calcium deposits in rat aortas stained with Alizarin Red. **(E)** Quantification of calcium deposits in rat aortas stained with Von Kossa. Six rats were included in each group. *P < 0.05 vs. sham-operated group; ^#^*P* < 0.05 vs. CRF group. CRF, chronic renal failure; HFD, high fat diet.

The aortic sections of rats in each group were stained with Alizarin Red, Von Kossa and HE staining at 6 months after operation to observe the aortic calcification. As shown in Figure 3A, the Alizarin Red staining revealed that there were no obvious calcium nodules in the aorta of the rats in sham-operated group, while the aorta of the rats in CRF and CRF + HFD groups showed scattered orange-red staining. The calcium nodules in the aorta of rats in CRF + HFD group were more obvious than those in CRF group. Von Kossa staining (Figure 3B) showed that there was a large amount of black granular calcium deposits between the elastic fibers of the aorta of the rats in CRF and CRF + HFD groups, and the calcium deposits in the aorta of the rats in CRF + HFD group were more significant than those in CRF group. But there was no obvious calcium deposition among the elastic fibers of the aorta in the sham-operated group. The HE staining showed that the aortic intima of rats in CRF and CRF + HFD groups was shrunken and broken and the nuclear arrangement was disordered, while the aortic intima of the sham-operated group was flat. The nuclei are arranged neatly (Figure 3C).

### Comparison of BMP-2 mRNA and Protein Expression in the Aorta of Rats in Each Group

**Figure 4:**
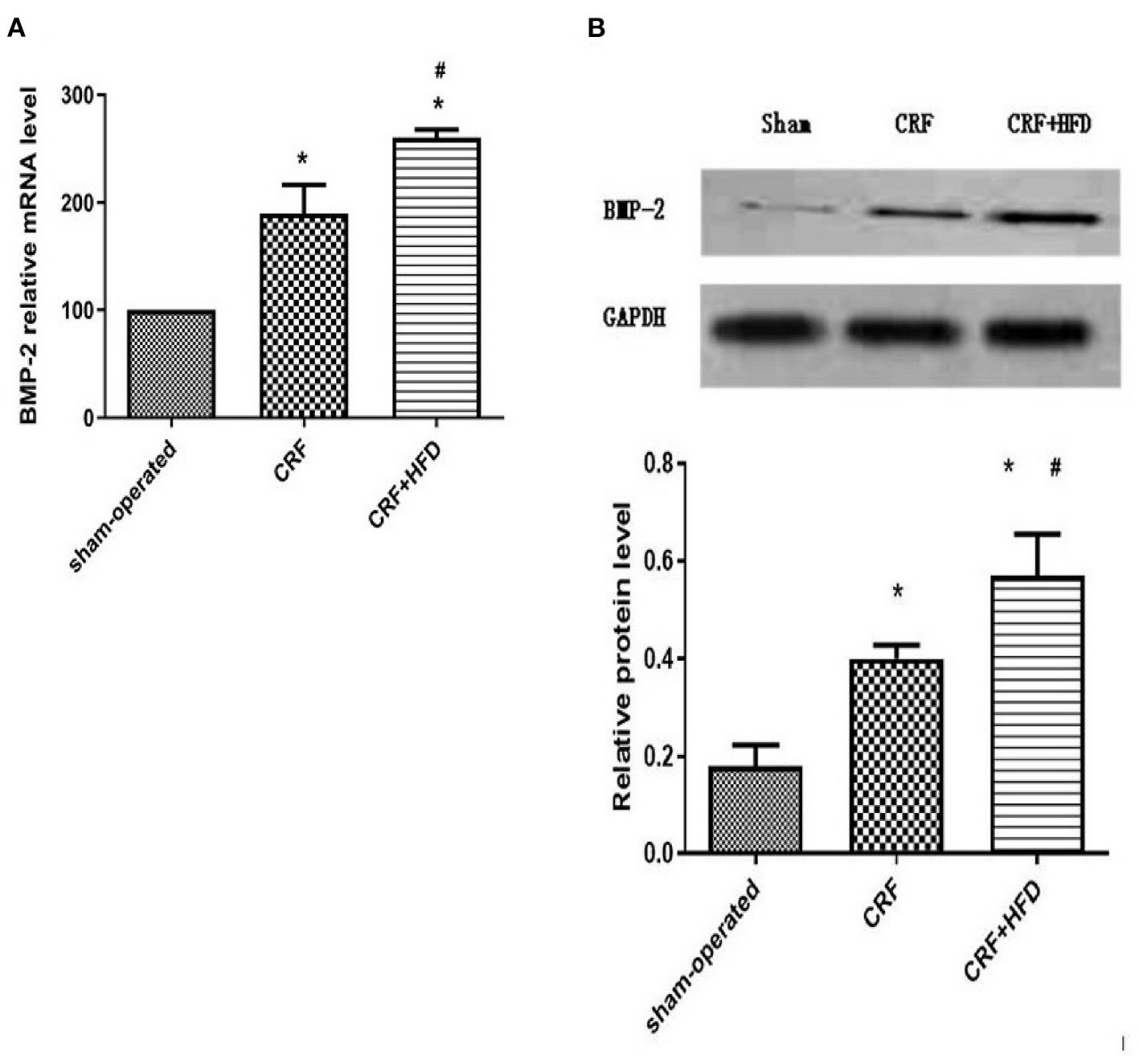
Comparison of BMP-2 mRNA **(A)** and protein **(B)** expression in the aortas of rats in each group. Six rats were included in each group. *P < 0.05 vs. sham-operated group; ^#^*P* < 0.05 vs. CRF group. BMP-2, bone morphogenetic protein-2; CRF, chronic renal failure; HFD, high fat diet.

The rat aortas of each group were taken 6 months after the operation to detect the expression of BMP-2 mRNA and protein. The results showed that compared with the sham-operated group, the BMP-2 mRNA and protein expression of the aortas in the CRF and CRF + HFD groups were significantly increased (*p* < 0.05, Figure 4A). Moreover, the expression of BMP-2 mRNA and protein in the aortas of the CRF + HFD group was significantly higher than that of the CRF group (*p* < 0.05, Figure 4B).

### Immunohistochemical Staining of BMP-2 Protein Expression in Aortas of Rats in Each Group

**Figure 5:**
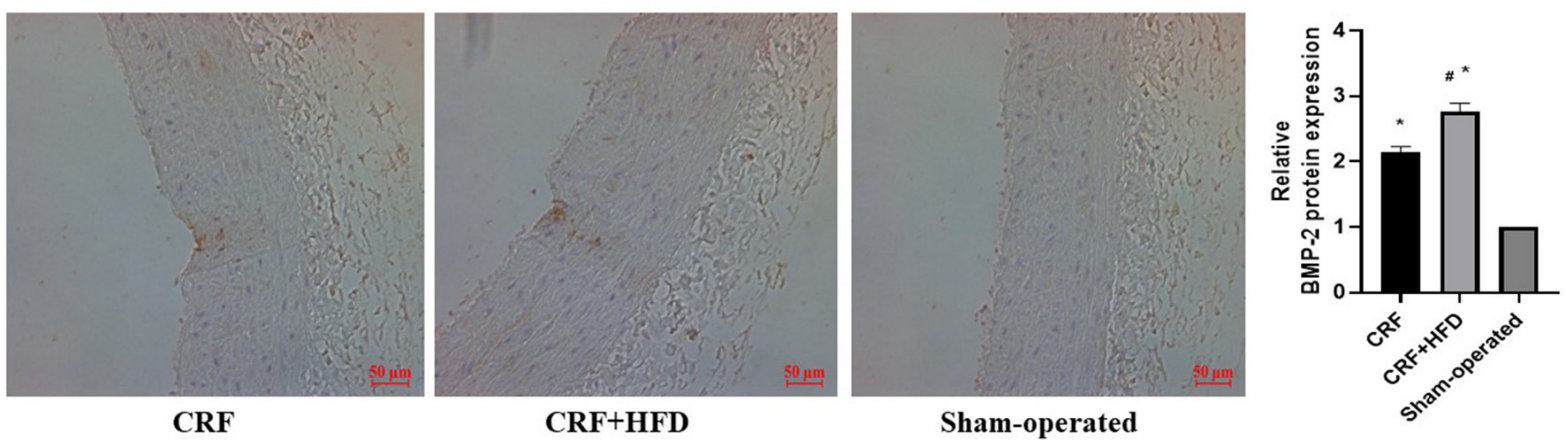
Immunohistochemical staining of BMP-2 protein expression in aortas of rats in each group. Six rats were included in each group. The BMP-2 protein staining of the aortic wall was light brown fine granular. *P < 0.05 vs. sham-operated group; ^#^*P* < 0.05 vs. CRF group. CRF, chronic renal failure; HFD, high fat diet.

The aortas of each group were separated 6 months after operation, and routine paraffin sections were used to observe the expression of BMP-2 protein in the aortic wall by immunohistochemical staining (Figure 5). The results revealed that the BMP-2 protein staining of the aortic wall in the CRF and CRF + HFD groups was light brown fine granular, and the BMP-2 protein expression in the aorta of the CRF + HFD group was more obvious than that of the CRF group. While there was no obvious BMP-2 protein expression in the aortic wall of the sham-operated group.

### Trend of Serum IL-18 in Rats of Each Group

**Figure 6:**
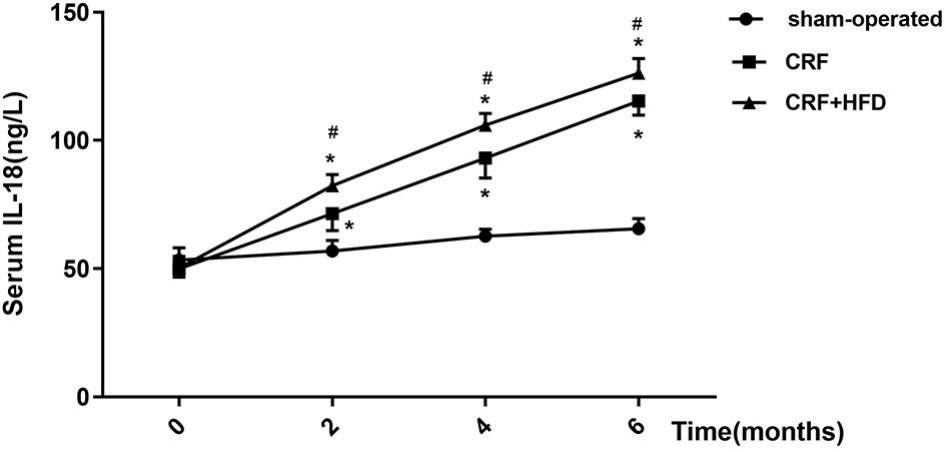
Trend of serum IL-18 in rats of each group after operation. Six rats were included in each group. *P < 0.05 vs. sham-operated group; ^#^*P* < 0.05 vs. CRF group. CRF, chronic renal failure; HFD, high fat diet.

The venous blood of each group was collected before operation and the second, fourth, and 6th month after the operation, and the serum IL-18 expression level was detected by ELISA (Figure 6). The results suggested that the postoperative serum IL-18 level of rats in the CRF and CRF + HFD groups gradually increased with time, while the postoperative serum IL-18 level of rats in the sham-operated group did not change significantly. Moreover, the postoperative serum IL-18 levels of rats in the CRF and CRF + HFD groups were significantly higher than those in the sham-operated group (*p* < 0.05). In addition, the serum IL-18 level in the CRF + HFD group was significantly higher than that in the CRF group (*p* < 0.05).

### Correlation Analysis of Rat Serum IL-18 Level and Aortic Calcification Content

**Figure 7:**
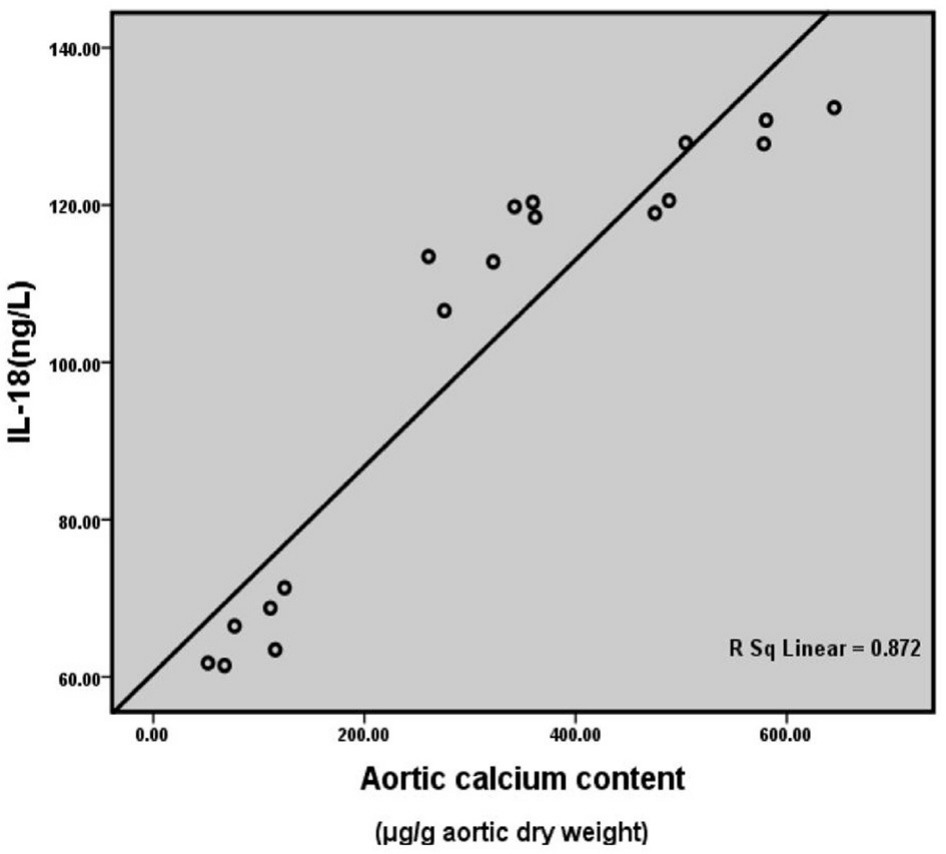
Correlation analysis of rat serum IL-18 level and aortic calcification content. Six rats were included in each group.

Correlation analysis between serum IL-18 level and aortic calcium content of all the rats in three groups at the 6th month after surgery (Figure 7) showed that serum IL-18 level was positively correlated with aortic calcium content (*r* = 0.934, *p* < 0.001), indicating that with the increase of serum IL-18 level, the degree of aortic calcification in rats became serious.

### The Activation of IL-18 on MAPK Pathways

**Figure 8:**
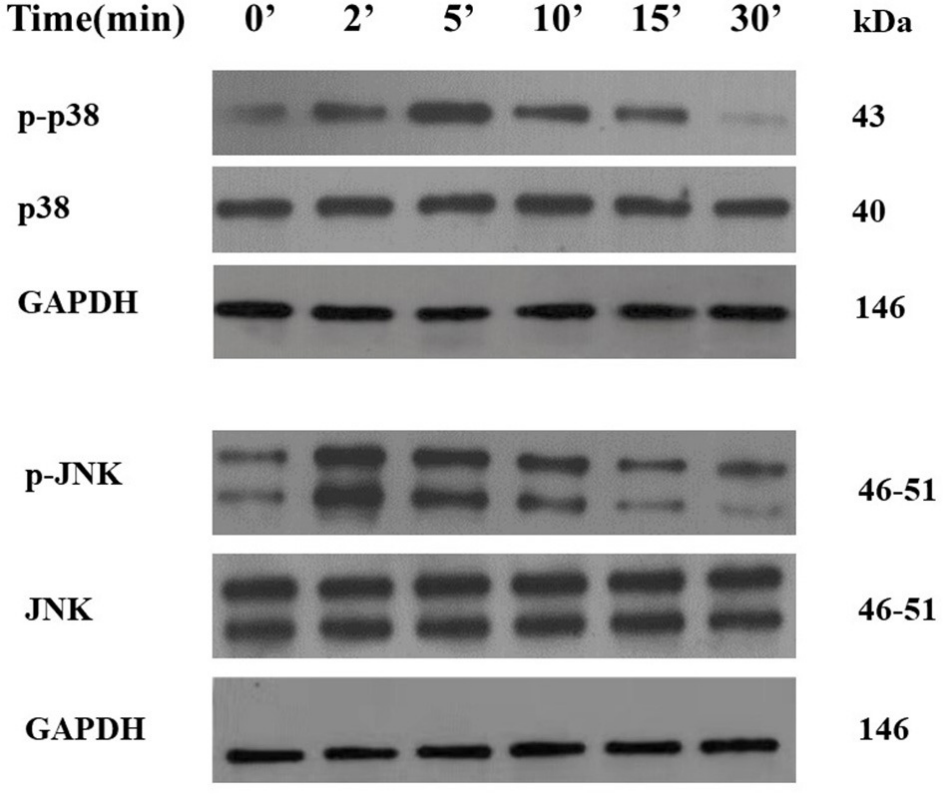
Activation of p38 and JNK pathways by IL-18 in rat VSMCs. After using IL-18 to interfere with rat VSMCs for 2, 5, 10, 15, and 30, the phosphorylation and non-phosphorylation protein expression levels of two MAPK pathways including p38 and JNK were detected by Western blot. The experiment was repeated 3 times independently.

As shown in Figure 8, when IL-18 intervened for 2 min, the expression of phosphorylated p38 (p-p38) protein in VSMCs began to increase. The expression of p-p38 was the most significant when IL-18 intervened for 5 min. After 10 min of IL-18 intervention, the expression of p-p38 began to show a downward trend. Moreover, 2 min after IL-18 treatment, p-JNK protein significantly increased. After IL-18 acted for 5 min, the protein expression of p-JNK showed a gradually decreasing trend.

### Effect of p38 Pathway on IL-18-Induced Calcification and Osteogenic Phenotypic Transformation of Rat VSMCs

**Figure 9:**
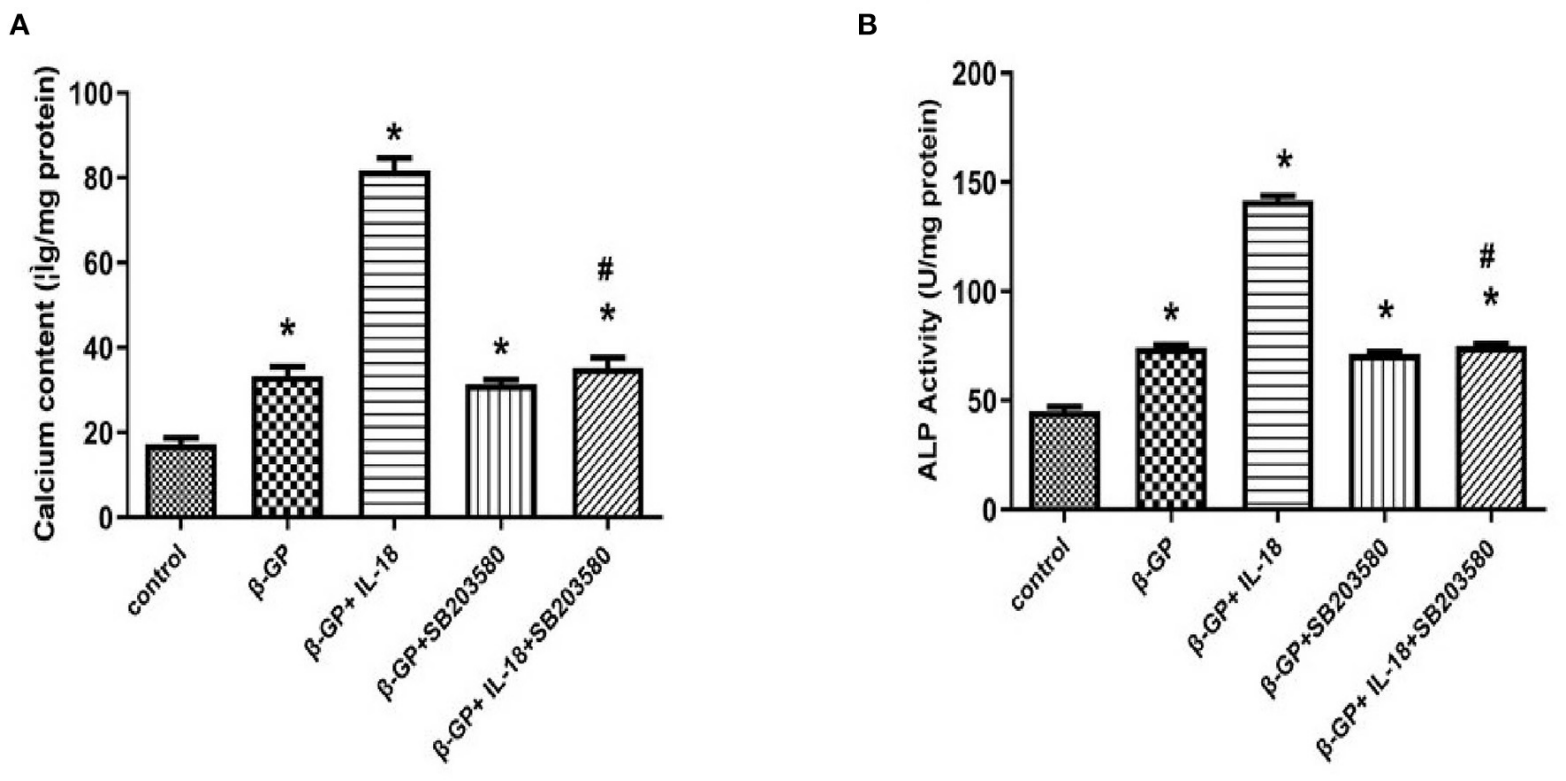
Effect of p38 pathway on IL-18-induced calcification of rat VSMCs. **(A)** Effect of p38 pathway on calcium content of rat VSMCs after IL-18 intervention. **(B)** Effect of p38 pathway on ALP activity of rat VSMCs after IL-18 intervention. Rat VSMCs were pre-incubated with p38 inhibitor SB203580 (10 μmol/L) for 2 h, and then treated with β-GP (10 mmol/L) or IL-18 (100 ng/ml) + β-GP (10 mmol/L) for 14 days, respectively. The experiment was repeated 3 times independently. *P < 0.01 vs. control group; ^#^*P* < 0.01 vs. IL-18 + β-GP group. ALP, alkaline phosphatase; BMP-2, bone morphogenetic protein-2; β-GP, β-glycerol phosphate.

**Figure 10:**
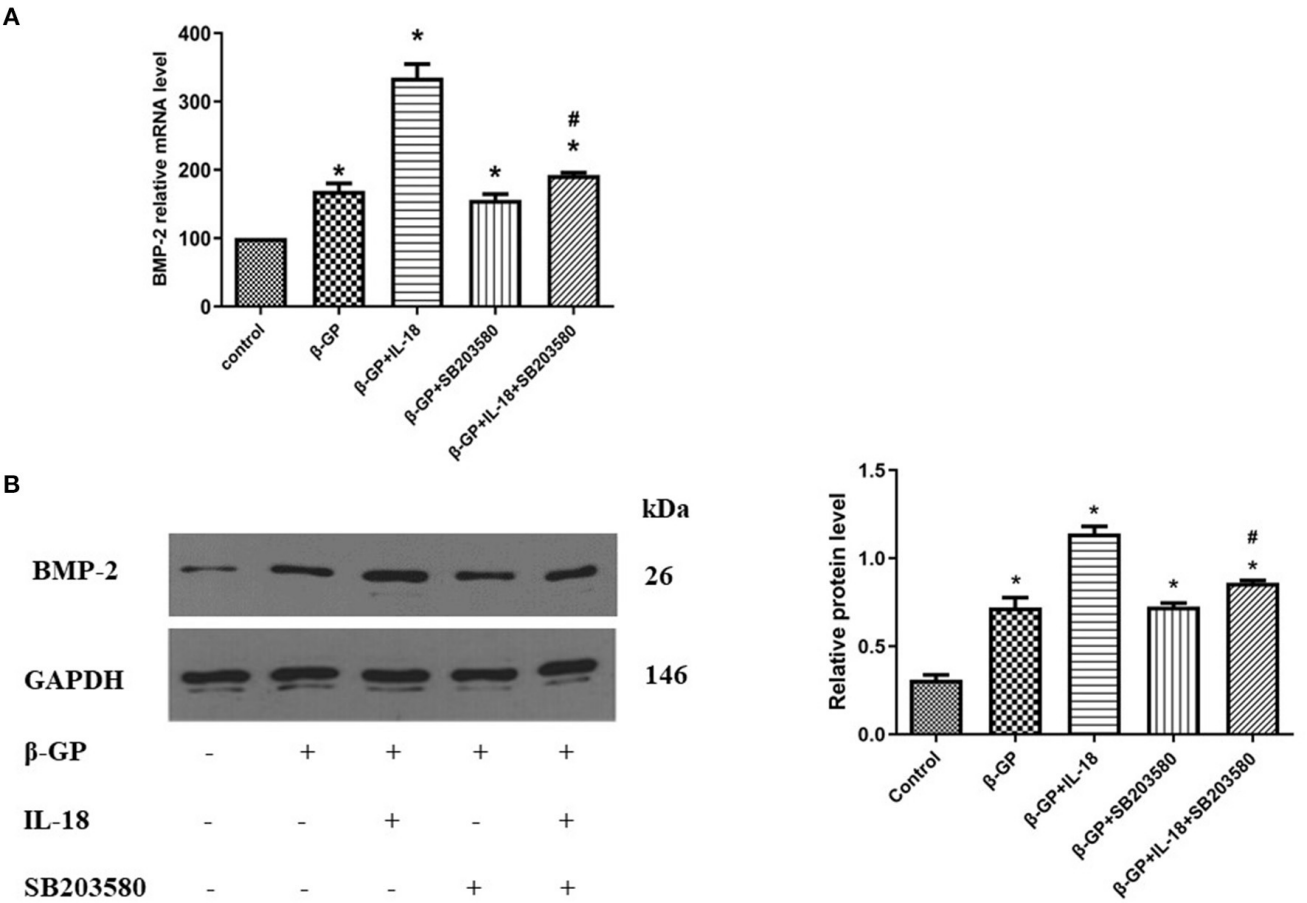
Effect of p38 pathway on IL-18-induced osteogenic phenotypic transformation of rat VSMCs. **(A)** Effect of p38 pathway on the BMP-2 mRNA expression of rat VSMCs after IL-18 intervention. **(B)** Effect of p38 signal pathway on the BMP-2 protein expression of rat VSMCs after IL-18 intervention. Rat VSMCs were pre-incubated with p38 inhibitor SB203580 (10 μmol/L) for 2 h, and then treated with β-GP (10 mmol/L) or IL-18 (100 ng/ml) + β-GP (10 mmol/L) for 14 days, respectively. The experiment was repeated 3 times independently. *P < 0.01 vs. control group; ^#^*P* < 0.01 vs. IL-18 + β-GP group. BMP-2, bone morphogenetic protein-2; β-GP, β-glycerol phosphate.

**Figure 11:**
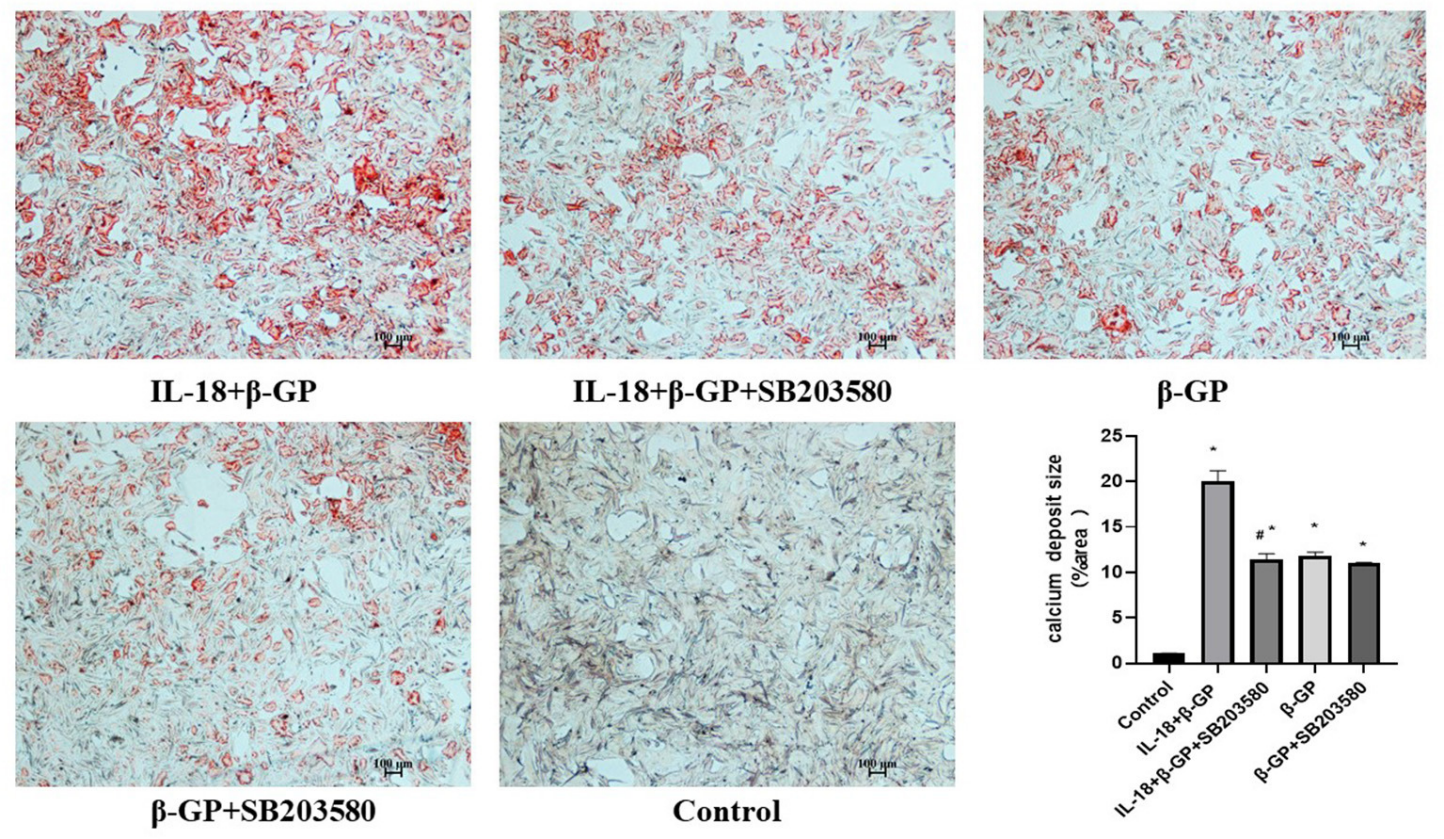
Alizarin red staining of VSMCs. Effect of p38 pathway on IL-18-induced calcium deposition of rat VSMCs. Rat VSMCs were pre-incubated with p38 inhibitor SB203580 (10 μmol/L) for 2 h, and then treated with β-GP (10 mmol/L) or IL-18 (100 ng/ml) + β-GP (10 mmol/L) for 14 days, respectively. The experiment was repeated 3 times independently. *P < 0.01 vs. control group; ^#^*P* < 0.01 vs. IL-18 + β-GP group. β-GP, β-glycerol phosphate.

We found that SB203580 decreased IL-18-enhanced calcium content (35.21 ± 3.46 vs. 81.83 ± 4.05, *p* < 0.01) and ALP activity (74.99 ± 1.63 vs. 141.75 ± 3.32, *p* < 0.01)in the IL-18 + β-GP group (Figure 9). Furthermore, SB203580 decreased IL-18-enhanced the expression of BMP-2 mRNA and protein in the IL-18 + β-GP intervention group (*p* < 0.01, Figure 10). But SB203580 pre-incubation had no significant effect on the calcium content (31.42 ± l.50 vs. 33.38 ± 2.91, *p* > 0.05) and ALP activity (71.32 ± l.53 vs. 74.06 ± 2.29, *p* > 0.05) of the β-GP group (Figure 9). SB203580 pre-incubation had no significant effect on the expression of BMP-2 mRNA and protein in the β-GP group (*p* > 0.05, Figure 10). Alizarin Red staining showed that SB203580 pre-incubation can significantly reduce the calcium deposition of IL-18 + β-GP group. The β-GP group pre-incubated with SB203580 and the β-GP group not pre-incubated with SB203580 showed no significant changes in cellular calcium deposition (Figure 11).

### Effect of JNK Pathway on IL-18-Induced Calcification and Osteogenic Phenotypic Transformation of Rat VSMCs

**Figure 12:**
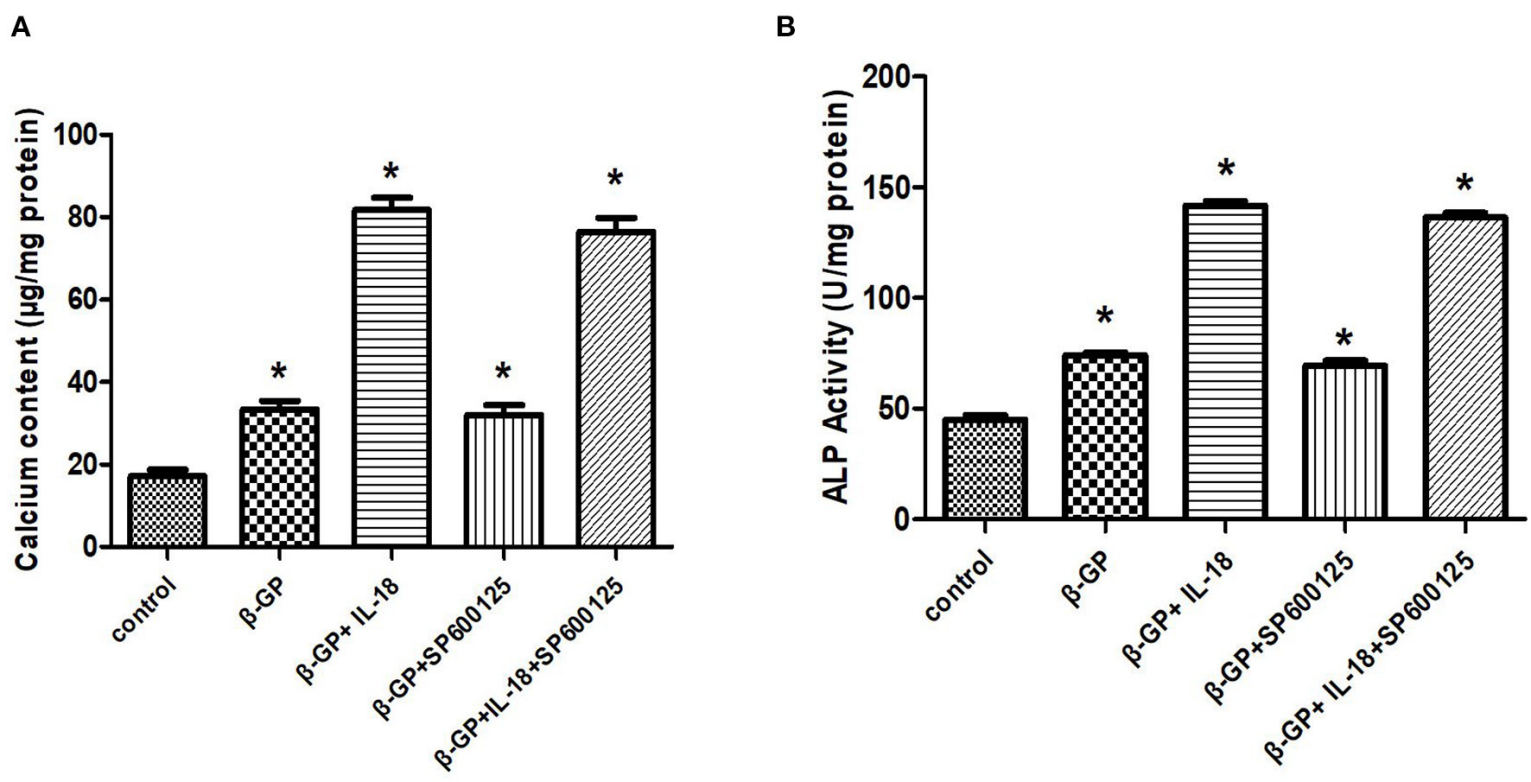
Effect of JNK pathway on IL-18-induced calcification of rat VSMCs. **(A)** Effect of JNK pathway on calcium content of rat VSMCs after IL-18 intervention. **(B)** Effect of JNK pathway on ALP activity of rat VSMCs after IL-18 intervention. Rat VSMCs were pre-incubated with JNK inhibitor SP600125 (10 μmol/L) for 30 min, and then treated with β-GP (10 mmol/L) or IL-18 (100 ng/ml) + β-GP (10 mmol/L) for 14 days, respectively. The experiment was repeated 3 times independently. *P < 0.01 vs. control group. ALP, alkaline phosphatase; BMP-2, bone morphogenetic protein-2; β-GP, β-glycerol phosphate.

**Figure 13:**
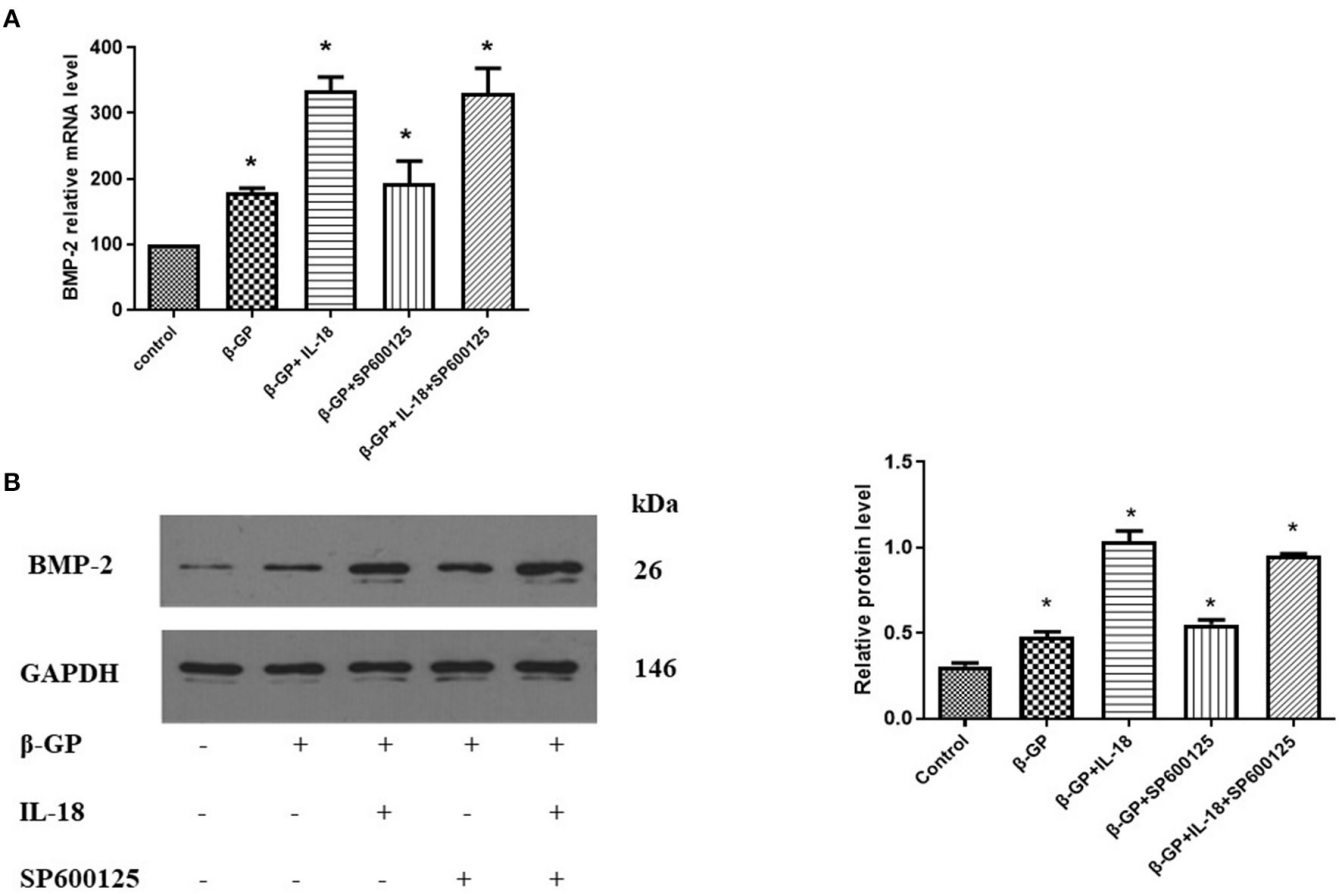
Effect of JNK pathway on IL-18-induced osteogenic phenotypic transformation of rat VSMCs. **(A)** Effect of JNK pathway on the expression of BMP-2 mRNA of rat VSMCs after IL-18 intervention. **(B)** Effect of JNK pathway on the expression of BMP-2 protein of rat VSMCs after IL-18 intervention. Rat VSMCs were pre-incubated with JNK inhibitor SP600125 (10 μmol/L) for 30 min, and then treated with β-GP (10 mmol/L) or IL-18 (100 ng/ml) + β-GP (10 mmol/L) for 14 days, respectively. The experiment was repeated 3 times independently. *P < 0.01 vs. control group. BMP-2, bone morphogenetic protein-2; β-GP, β-glycerol phosphate.

**Figure 14:**
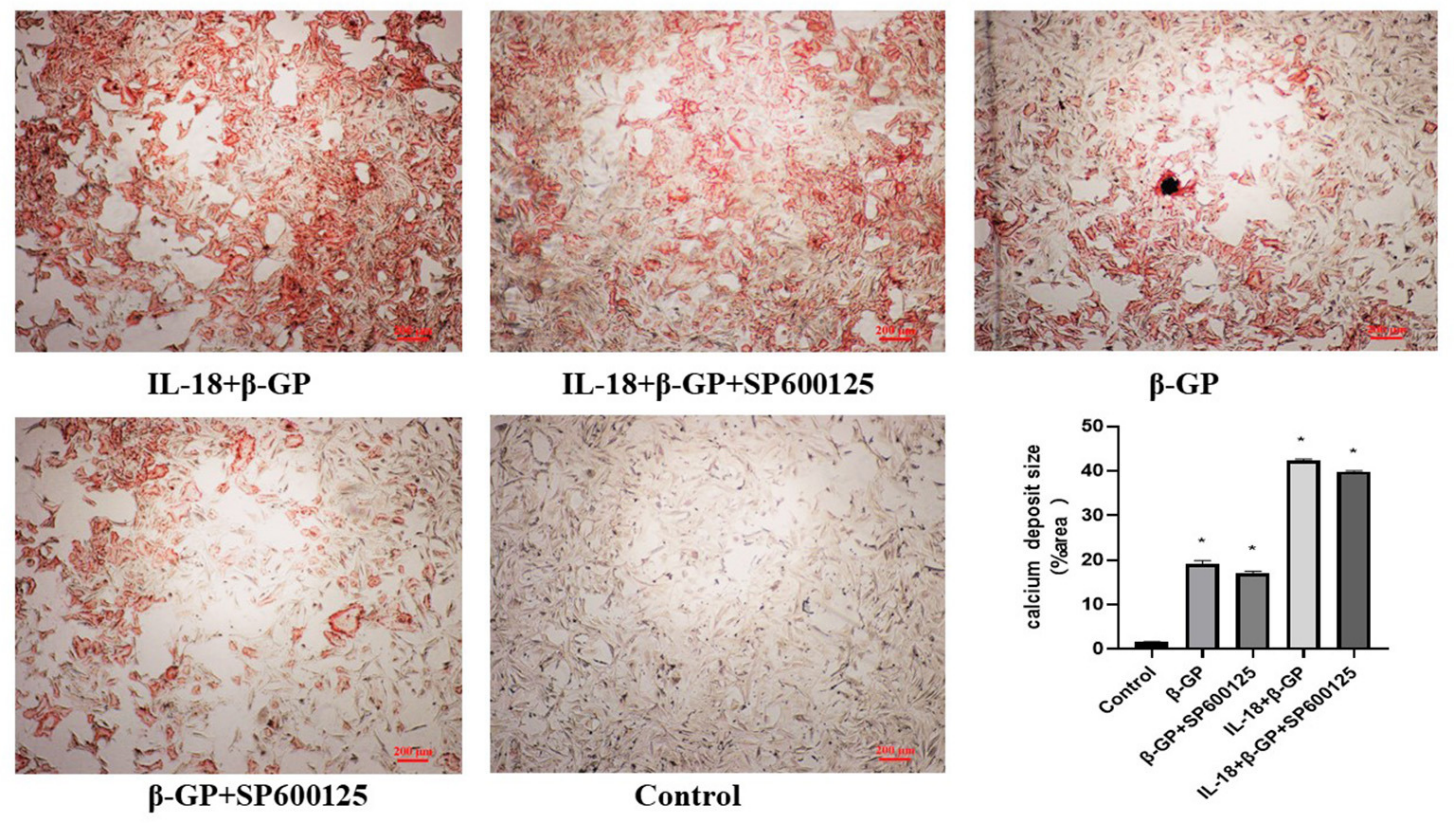
Alizarin red staining of VSMCs. Effect of JNK pathway on IL-18-induced calcium deposition of rat VSMCs. Rat VSMCs were pre-incubated with JNK inhibitor SP600125 (10 μmol/L) for 30 min, and then treated with β-GP (10 mmol/L) or IL-18 (100 ng/ml) + β-GP (10 mmol/L) for 14 days, respectively. And then the VSMCs were stained with Alizarin red. The experiment was repeated 3 times independently. *P < 0.01 vs. control group. β-GP, β-glycerol phosphate.

The results indicated (Figure 12) that SP600125 had no significant effect on IL-18-enhanced calcium content (76.42 ± 4.86 vs. 81.83 ± 4.05, *p* > 0.05) and ALP activity (136.36 ± 3.50 vs. 141.75 ± 3.32, *p* > 0.05) in the IL-18 + β-GP group. And SP600125 also had no significant effect on IL-18-enhanced expression of BMP-2 mRNA and protein in the IL-18 + β-GP group (*p* > 0.05, Figure 13). Furthermore, SP600125 pre-incubation had no significant effect on the calcium content (32.0 ± 3.32 vs. 33.38 ± 2.91, *p* > 0.05) and ALP activity (69.48 ± 3.96 vs. 74.06 ± 2.29, *p* > 0.05) of the β-GP group (Figure 12). SP600125 pre-incubation had no significant effect on the expression of BMP-2mRNA and protein in the β-GP group (*p* > 0.05, Figure 13). SP600125 pre-intervention has no significant effect on calcium deposition in IL-18 + β-GP and β-GP groups (*p* > 0.05, Figure 14).

## Discussion

VC is a preventable, reversible and highly adjustable process similar to bone and cartilage formation, and the key mechanism of VC is the osteogenic differentiation of VSMCs (25). Inflammation is currently considered to be one of the important factors regulating the development of VC (7). Our previous research results suggested that in subjects without CRF, obesity-related inflammation might be involved in regulating the formation of VC (15). Obesity conferred greater cardiovascular risk when combined with metabolic syndrome in CRF patients (26). VC is a common vascular complication of CRF and an important indicator of poor prognosis. Nowadays, the relationship between obesity-related inflammation and VC in CRF patients is still controversial. Krasniak et al. had proved that the coronary artery calcification score is positively correlated with BMI in maintenance haemodialysis patients (27). But the study by Kim et al. concluded that there was no significant correlation between obesity and aortic calcification in dialysis patients (28). Therefore, this study explored the effect of obesity caused by high-fat diet on VC in a rat model of CRF.

The results of the study showed that the calcium content and BMP-2 expression of the aorta in the CRF rats fed with high-fat diet were significantly higher than the CRF rats fed with conventional diet. This study confirms for the first time that in a rat model of CRF, feeding with high-fat diet can further induce the osteogenic phenotype transformation and deteriorate VC.

Indeed, it is now widely agreed that obesity is a state of low-grade chronic inflammation (29, 30). Increased circulating levels of inflammatory cytokines have been reported in overweight and obese adults, and this event has been linked to the increased cardiovascular risk seen in obesity (31). Our study also showed that the body weight, blood lipids and hsCRP level of CRF rats fed with high-fat diet were significantly higher than those of CRF rats fed with conventional diet. HsCRP is one of the important inflammatory factors, suggesting that in CRF rats, a high-fat diet may cause obesity-related inflammation.

Some studies have confirmed that IL-18 is an important inflammatory marker of MetS, which is involved in regulating the development of MetS. In addition, studies have also shown that IL-18 is closely related to obesity and is one of the important regulators of obesity-related inflammation (32, 33). Bruun and Jung et al. all proved that the IL-18 levels in adipose tissue and serum of obese subjects were significantly higher than those of control subjects (34, 35). Our previous study found that IL-18 can induce calcification of VSMCs (7). However, whether IL-18 is involved in the regulation of VC in CRF patients with obesity has not been reported yet. This study indicated that the serum IL-18 levels of CRF rats fed with high-fat diet and a regular diet both showed an increasing trend with time, and the serum IL-18 levels of these two groups were significantly higher than the sham-operated group. Moreover, the postoperative serum IL-18 level of CRF rats fed with high-fat diet was significantly higher than that of CRF rats fed with conventional diet, which indicated that obesity caused by high-fat diet can further upregulate serum IL-18 levels in CRF rats. We further analyzed the correlation between serum IL-18 level and VC. The results suggest that the serum IL-18 level of rats is positively correlated with the aortic calcium content, indicating that as the serum IL-18 level increases, the degree of aortic calcification is aggravated. However, the mechanism by which IL-18 regulates the formation of VC is not yet clear and needs to be further explored.

Previous studies have proved that in patients with CRF, the levels of serum CRP, IL-6 and other inflammatory factors are positively correlated with the degree of coronary artery calcification (27, 36). In addition, a large number of *in vivo* and *in vitro* studies have also shown that inflammatory factors can induce the transformation of VSMCs into osteoblast phenotypes, and then promote VSMCs calcification (37, 38). Therefore, inflammation is considered to be an important regulator of VC. In our study, it was proved that the up-regulation of inflammatory factor levels in CRF rats fed with high-fat diet had a close correlation with the development of VC. And this results further indicated that obesity-related inflammation induced by high-fat diet might be an important regulator of VC in CRF rats.

The MAPK signaling pathway is a type of serine/threonine protein kinase that exists widely in mammals, which can be activated by a series of extracellular signals or stimuli. The JNK and p38 pathways are two main members of the MAPK signaling pathway. Previous studies had proved that IL-18 could cause different biological effects by activating the JNK and p38 signaling pathways (21, 22, 39), suggesting that the MAPK pathways might be the important downstream pathways for IL-18. Our research also showed that IL-18 could promote the expression of JNK and P38 pathway phosphorylated proteins in rat VSMCs, indicating that IL-18 could induce biological effects by activating the MAPK pathways in rat VSMCs. The results of Takahisa et al. showed that advanced glycation end products could promote VSMCs calcification by activating the p38 pathway (40). Our research also found that the p38 signaling pathway blocker SB203580 can significantly reduce the calcium content, ALP activity and BMP-2 expression of rat VSMCs induced by IL-18. The P38 signaling pathway is not only involved in the regulation of cell proliferation and survival (41), but also plays a key role in immune and inflammatory responses (42). The above results indicated that the p38 signal transduction pathway is also involved in the regulation of IL-18 to promote the process of osteoblast differentiation and calcification of VSMCs. OPG is one of the osteoblast transcription factors closely related to VC. The study by McCarthy et al. found that the effect of PDGF on osteoblast cell lines to produce OPG is regulated by p38 signaling pathways, but not affected by the JNK signaling pathway (43). Although the results of this study found that IL-18 can also activate the JNK signaling pathway, blocking the JNK pathway has no significant effect on VSMCs calcification induced by IL-18, which suggested that the JNK signaling pathway might not be involved in regulation the process of IL-18-induced VC. Although different MAPK pathways of have similar cascade reactions, the biological effects produced by different extracellular activation signals are not completely the same.

In summary, it was demonstrated that obesity-related inflammation induced by high-fat diet could elevate serum IL-18 levels and exacerbate VC in CRF rats. Furthermore, serum IL-18 level had a positive correlation with the degree of VC. It is also found that IL-18 promoted osteogenic differentiation and calcification of rat VSMCs via p38 pathway activation.

## Data Availability Statement

The raw data supporting the conclusions of this article will be made available by the authors, without undue reservation.

## Ethics Statement

The animal study was reviewed and approved by Committee on Ethics of Animal Experiments of Sun Yat-sen University.

## Author Contributions

YiZ is mainly responsible for experimental design and article writing. HH and JW are responsible for guiding the implementation of the research and the revision of the article. KZ and LZ is responsible for the implementation of animal experiments. YuZ is responsible for data statistical analysis and article revision. All authors contributed to the article and approved the submitted version.

## Funding

This work was supported by National Natural Science Foundation of China (81900443), Fund for Basic and Applied Basic Research of Guangdong Province (2018A030313749) and PhD Natural Sciences Startup Foundation of Guangdong (2017A030310230) to YiZ.

## Conflict of Interest

The authors declare that the research was conducted in the absence of any commercial or financial relationships that could be construed as a potential conflict of interest.

## Publisher's Note

All claims expressed in this article are solely those of the authors and do not necessarily represent those of their affiliated organizations, or those of the publisher, the editors and the reviewers. Any product that may be evaluated in this article, or claim that may be made by its manufacturer, is not guaranteed or endorsed by the publisher.

## Abbreviations

ALP: alkaline phosphatase
Apo: apolipoprotein
BMI: body mass index
BMP-2: bone morphogenetic protein-2
BSA: bovine serum albumin
Ca: calcium
CHE: cholinesterase
Cr: creatinine
CRF: chronic renal failure
CVD: cardiovascular disease
HDL-C: high-density lipoprotein cholesterol
LDL-C: low-density lipoprotein cholesterol
hsCRP: high-sensitivity C-reactive protein
MAC: medial arterial calcification
MAP: mean arterial pressure
MetS: metabolic syndrome
OB/MS: obesity and metabolic syndrome
OR: odds ratio
P: phosphate
PVDF: polyvinylidene difluoride
SBP: systolic pressure
SD: standard deviation
SOD: superoxide dismutase
TC: total cholesterol
TG: triglycerides
UA: uric acid
VC: vascular calcification
VSMCs: vascular smooth muscle cells.
